# Impact of adapted physical activity on hippocampal N-Acetyl Aspartate in patients with schizophrenia

**DOI:** 10.1192/j.eurpsy.2023.961

**Published:** 2023-07-19

**Authors:** L. Metivier, F. Briend, M. Tréhout, L. Bigot, G. Quarck, A. Herbinet, E. Leroux, S. Dollfus

**Affiliations:** 1 Calvados, Normandie Univ, UNICAEN, PhIND, UMR-S 1237, GIP CYCERON, Caen; 2 Indre et Loire, UMR Inserm U 1253 – iBrain – Psychiatrie Neuro-Fonctionelle – Equipe 1, Tours; 3 Calvados, CHU de CAEN Normandie, Service de Psychiatrie, Centre Esquirol; 4 Calvados, Mooven; 5 Calvados, Normandie Univ, UNICAEN/INSERM, UMR 1075, COMETE, PFRS, Caen; 6 Hérault, CSO, Mooven, Saint-Mathieu-de-Treviers, France

## Abstract

**Introduction:**

Adapted physical activity (APA) has beneficial neurobiological impact but the underlying pathophysiological mechanisms remain poorly described. APA is currently recognized as an adjuvant therapy to antipsychotic treatments in patients with schizophrenia (SCZs) to reduce the severity of negative symptoms and cognitive impairment. SCZs exhibit hippocampal N-acetylaspartate (NAA) reduction, a marker of neuronal viability and integrity whose concentrations can be assessed by proton magnetic resonance spectroscopy (^1^H-MRS).

**Objectives:**

The purpose of this study was to evaluate the impact of remote physical activity (e-APA) via the web on the NAA relative variations in the left hippocampus in SCZs compared to a patient control group benefiting from an health education program (HE). This study concerns one of the secondary objectives of the PEPsy V@SI study co-financed by the Pierre Deniker Foundation, the European Union and the Normandy Region within the framework of the FEDER/FSE 2014-2020 operational program.

**Methods:**

Thirty-five SCZs were randomized in the e-APA active group or in the control group (HE). Participants received the interventions during 16 weeks, with two visioconference sessions per week. A ^1^H-MRS sequence positioned on the left hippocampus (MRI-3T) was acquired before and after both interventions. Absolute NAA concentrations in the left hippocampus were obtained using Osprey software after partial volume correction. After checking the quality criteria, the spectra of 6 SCZs in the e-APA group and 8 SCZs in the HE group were analyzed. To test the difference between interventions on the NAA relative variations, a Wilcoxon-Mann-Whitney test and effect size were performed. Paired Wilcoxon tests were used in each group before and after the interventions.

**Results:**

No significant difference was found in NAA relative variations in the left hippocampus between the e-APA group and the HE group (p = 0.18), although the effect size was 0.38 (considered as moderate). However, a trend towards an increase of NAA was observed in the e-APA group (before intervention: 12.08 International Units (I.U); after: 13.81 I.U) (p = 0.06) but not in the HE group (before intervention: 13.75 I.U ; after: 13.85 I.U) (p = 0.84).

**Conclusions:**

Our results showed a NAA significant increase in SCZs after an e-APA program, indicating a beneficial impact of e-APA on neuronal viability that might reflect an hippocampal plasticity. However, this increase did not differ significantly between active and control groups probably due to a weak statistical power.

**Disclosure of Interest:**

L. Metivier: None Declared, F. Briend: None Declared, M. Tréhout: None Declared, L. Bigot: None Declared, G. Quarck: None Declared, A. Herbinet: None Declared, E. Leroux: None Declared, S. Dollfus Consultant of: Fabre,Gedeon,Roche and Takeda, inivited Conferences by Lundbeck, Otsuka, Janssen ; at contracts with Prophase MedAvances and NeuroCogTrials

